# Large language models know how the personality of public figures is perceived by the general public

**DOI:** 10.1038/s41598-024-57271-z

**Published:** 2024-03-20

**Authors:** Xubo Cao, Michal Kosinski

**Affiliations:** Stanford University, Stanford, USA

**Keywords:** Psychology, Computer science

## Abstract

We show that people’s perceptions of public figures’ personalities can be accurately predicted from their names’ location in GPT-3’s semantic space. We collected Big Five personality perceptions of 226 public figures from 600 human raters. Cross-validated linear regression was used to predict human perceptions from public figures’ name embeddings extracted from GPT-3. The models’ accuracy ranged from r = .78 to .88 without controls and from r = .53 to .70 when controlling for public figures’ likability and demographics, after correcting for attenuation. Prediction models showed high face validity as revealed by the personality-descriptive adjectives occupying their extremes. Our findings reveal that GPT-3 word embeddings capture signals pertaining to individual differences and intimate traits.

## Introduction

People’s success and well-being heavily depend on how their personalities are judged by others and—increasingly—algorithms^1^. Ranging from the first impressions based on facial appearance^2^ to close friends’ well-informed opinions^3^, others’ perceptions affect one’s personal, educational, and occupational success; social capital; health; wealth; and many other consequential outcomes^4^. Importantly, others’ perceptions matter regardless of their accuracy^3,5^, as illustrated by those suffering (or benefiting) from prejudice and stereotypes^6,7^.

Particularly consequential are perceptions of public figures’ personalities. Politicians’ perceived personality influences their electoral success^8^, their approval ratings^9^, and even geopolitics^10^. CEOs’ perceived personality influences their own success but also their companies’ reputation, valuation, and performance^11,12^. Celebrities’ perceived personality affects the recognition, consumer attitudes, and purchase intentions toward the brands they promote^13^. Musicians’ perceived personality drives their music’s popularity^14^. Unsurprisingly, public figures invest much effort and resources into shaping others’ impressions, while researchers and practitioners across many disciplines study their formation and assessment^15,16^.

Perceptions of public figures’ personalities are typically measured by surveying qualified informants or the general public, a costly and time-consuming approach^9,12^. Such perceptions are also reflected in public discourse and communications^17^. As public discourse and journalism increasingly shift to digital environments, people’s views and perceptions are now increasingly recorded in written digital sources such as blog posts, tweets, Wikipedia entries, newspaper articles, and books. This signal is further amplified, as people’s perceptions and actual personality cues shape others’ perceptions, leading to self-amplifying feedback loops. Taken together, these phenomena suggest that costly and time-consuming surveys could be supplemented with perceived personality estimates extracted from digital language samples.

Past research has confirmed that perceptions of others’ personalities could be successfully extracted from texts, such as social media posts, biographies, or books^18–20^. The main challenge of this approach is obtaining the text corpora necessary to extract personality perception cues. Yet, this challenge has been addressed by the recent explosion in the size and availability of large language models (LLMs), such as Word2Vec, BERT, or GPT3^21–23^. LLMs are trained on huge and diverse text corpora that include, among other things, language revealing people’s perceptions of public figures’ personalities as well as the cues to their actual personalities. For example, GPT-3—the state-of-the-art LLM model used here—was trained on the contents of billions of websites, the entire English Wikipedia, and over 10,000 books^23^. The collection and analysis of such data are beyond the technological capacity of most researchers, not to mention the associated financial and environmental costs. For example, the training of GPT-3 was estimated to cost $12 million and to emit 552 tons of carbon dioxide^24,25^.

Past work showed that the perceptions of public figures’ warmth and competence can be extracted from an earlier LLM, Google’s Word2Vec^26^. Here we show that word embeddings extracted from GPT-3^23^ can predict people’s general sentiment toward public figures (*likability*) as well as their perceptions of their Big Five personality traits (*openness*, *conscientiousness*, *extraversion*, *agreeableness*, and *emotional stability*) that were shown to capture much variance in individual differences and reliably predict a wide range of individual and social outcomes^27^.

## Methods

Our study focused on the 300 most popular public figures from 43 countries selected from among the 11,341 public figures listed in the Pantheon 1.0 dataset^28^. Their popularity was approximated by their Wikipedia page views between 2008 and 2013. As artists were particularly popular, we limited their number to 100 to include public figures from seven other domains including *business and law*, *exploration*, *humanities*, *institutions*, *science and technology*, *sports*, and *others.* The dataset includes public figures’ gender and birth year (with some missing data). As raters may have been less familiar with public figures born before 1900, we did not include them in our studies.

Public figures’ names were presented to raters employed on Prolific. Each of the 600 raters rated the likability (on a 200-point scale from extremely negative to extremely positive) and Big Five personality traits (on the Ten-Item Personality Inventory; TIPI) of 10 random public Figures^29^. Raters could skip targets that they were unfamiliar with. Public figures received 18.89 ratings on average (SD = 10.38). We removed 74 public figures who were recognized (and thus rated) by fewer than 10 raters. See Supplementary Materials for the rationale and the intraclass correlation coefficient (ICC)^30^, a measure of the agreement between two or more raters.

GPT-3 stores knowledge about words’ meaning in a 12,288-dimensional semantic space, a functional equivalent of the semantic memory in humans. The closer two words (or phrases) are in this space, the more similar their meaning^31^. For example, “Donald Trump” is similar to “arrogant,” while “Mother Teresa” is close to “sympathetic.” The embeddings of public figures’ names, representing their location in this space, were entered into a Ridge regression^26^ to predict human ratings. Ridge regression is suitable for the analyses of high-dimensional data, as it reduces multicollinearity between predictors by penalizing large coefficients. The embeddings were standardized (by column) to ensure that the penalty was applied equally to each dimension. To prevent overfitting, 20-fold cross-validation was used: Predictions for each public figure were estimated using a model trained on all other public figures. The alpha parameter was tuned within each cross-validation fold using another 20-fold cross-validation.

Like all measures, human ratings include some errors. The split-half reliability of the ratings for the six attributes that we measured ranges from 0.79 to 0.88. This range serves as a benchmark for the highest accuracy that a predictive model might potentially achieve. Given that our interest lies in accurately predicting actual perceived personalities—not imperfect proxies—we adjusted the correlations using the square root of each scale’s reliability, a process known as correction for attenuation^32^. This adjustment enables a more equitable comparison of the model’s performance across various traits, notwithstanding the differing levels of agreement among human raters about these traits. For transparency, we also report the raw, uncorrected values.

## Results

**Figure 1:**
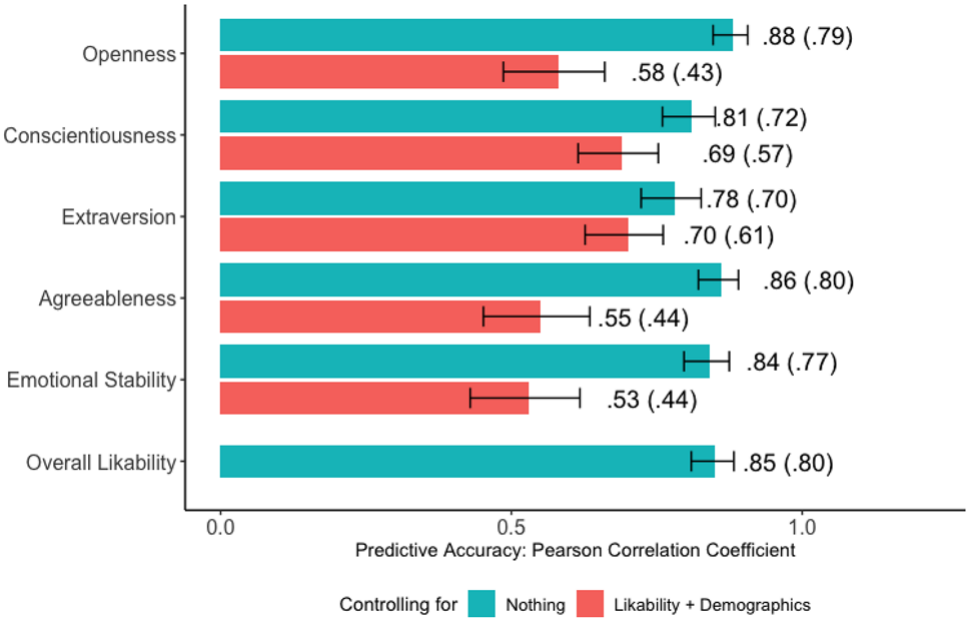
The model’s accuracy in predicting public figures’ perceived personality without any controls (green bars) and while controlling for likability and demographics (red bars). Confidence intervals equal 95%. Values in parentheses represent raw accuracy (uncorrected for attenuation). All correlations are significant at the p < .001 level.

Figure 1 (green bars) shows that GPT-3’s embeddings accurately predicted human perceptions. The Pearson product-moment correlations between models’ predictions and human ratings ranged from r = 0.78 for extraversion to r = 0.88 for openness, which translates into Cohen’s d range of d = 2.49 (huge effect) to d = 3.75 (huge effect)^33^. Raw accuracy (i.e., the accuracy obtained without controlling for attenuation) was also high, ranging from r = 0.7 to r = 0.8. To put models’ accuracy in perspective, consider the following well-known diagnostic accuracies: The accuracy of computer tomography when detecting metastases from head and neck cancer equals r = 0.64, and the accuracy of ultrasonography when detecting peripheral artery disease is r = 0.83^34^. In other words, public figures’ perceived personalities can be inferred from the GPT-3 embeddings of their names with an accuracy comparable to how some of their ailments could be diagnosed by modern medical diagnostic tools. Moreover, given that individual human ratings predicted aggregate ratings with an accuracy of r = [0.56—0.66], embeddings predict aggregate judgments better than individual judgments do.

Predictions were more accurate for more popular public figures. The profile similarity between human ratings and model predictions was correlated with the logarithm of the number of Wikipedia pageviews at the level of r = 0.16 (refer to Supplementary Materials for more details). This indicates that it was more accurate for more popular figures that, presumably, appeared more frequently in its training data.

Table 1 shows the top and bottom 10 public figures, arranged according to their predicted perceived personality traits (full list at https://osf.io/854w2). It shows that the embedding-based predictions have high face validity. For example, individuals predicted to be perceived as the most open-minded, liberal, creative, and artistic (i.e., high openness) included mostly artists: Freddie Mercury, David Bowie, Michael Jackson, Lady Gaga, Jimi Hendrix, and Quentin Tarantino. In contrast, those predicted to be perceived as the most conservative and traditional (i.e., low openness) included mostly autocrats (Kim Jong-un and Kim Jong-il); conservative politicians (Richard Nixon, Mitt Romney, George H. W. Bush, Margaret Thatcher, Donald Trump, Dick Cheney, and George Bush); and Queen Elizabeth II.

**Table 1:** Top and bottom 10 public figures according to their predicted perceived traits.

	Public figures	
	Bottom (ascending)	Top (descending)
Agreeableness	Kim Jong-il Osama bin Laden Saddam Hussein Donald Trump Kim Jong-un Zodiac Killer Vladimir Putin Charles Manson Simon Cowell Lee Harvey Oswald	Pope John Paul II Steve Irwin Audrey Hepburn Anne Frank Julia Child Joseph Gordon-Levitt Mother Teresa Jacqueline Kennedy Onassis Ryan Reynolds Emma Watson
Conscientiousness	Charlie Sheen Donald Trump Bam Margera Charles Manson Amy Winehouse Lindsay Lohan O. J. Simpson Kurt Cobain Kanye West James Franco	Serena Williams Bruce Lee Nelson Mandela Warren Buffett Neil Armstrong Jackie Chan Yao Ming Stephen Hawking Julia Child Bill Gates
Emotional Stability	Zodiac Killer Charles Manson Donald Trump Lee Harvey Oswald Jeffrey Dahmer Kim Jong-un Jim Jones O. J. Simpson Saddam Hussein Kanye West	Pope John Paul II Nelson Mandela Bruce Lee Barack Obama Mother Teresa Bear Grylls Joseph Gordon-Levitt Jackie Chan Indira Gandhi Jimmy Carter
Extraversion	Mark Zuckerberg Lee Harvey Oswald Kristen Stewart Alan Turing Elizabeth II of the United Kingdom Jeffrey Dahmer Yao Ming Zodiac Killer Howard Hughes Stephen King	Steve Irwin Bam Margera Jim Carrey Dennis Rodman Conan O’Brien Nicki Minaj Hulk Hogan Miley Cyrus Oprah Winfrey Chris Jericho
Openness	Kim Jong-un Kim Jong-il Richard Nixon Mitt Romney George H. W. Bush Margaret Thatcher Elizabeth II of the United Kingdom Donald Trump Dick Cheney George Bush	Freddie Mercury David Bowie Michael Jackson Lady Gaga Jimi Hendrix Sir Richard Branson Bam Margera Julia Child Quentin Tarantino Steve Irwin
Likability	Zodiac Killer Ted Bundy Jim Jones Osama bin Laden Jeffrey Dahmer Kim Jong-il Saddam Hussein Lee Harvey Oswald Charles Manson Kim Jong-un	Anne Frank Rosa Parks Julia Child Steve Irwin Nelson Mandela Jackie Robinson Joseph Gordon-Levitt Audrey Hepburn George Orwell Simon Pegg

Full lists at https://osf.io/854w2.

A closer inspection of the names presented in Table 1 suggests a link between models’ predictions and public figures’ profession. Gender seems to play a role, too, with women dominating the top of the perceived agreeableness ranking and entirely absent from its bottom. Moreover, personality perceptions are linked with likability: Many of the least likable figures (e.g., Lee Harvey Oswald, Charles Manson, and both Kims) appear repeatedly on the socially undesirable (i.e., low) extrema of the perceived personality trait. As detailed in Table 2, public figures’ likability, birth year, and gender significantly correlate with perceived personality in our sample. Birth year, for example, correlates strongly and negatively with both human ratings of extraversion (r = 0.35) and GPT-3’s prediction (r = 0.44).

**Table 2:** Means, standard deviations, and correlations between human judgments, model predictions, and demographic variables.

Variable	*M*	*SD*	1	2	3	4	5	6	7	8	9	10	11	12	13
1. Birth year	1958.87	23.74													
2. Female	0.27	0.44	0.26**												
3. Likability	29.02	39.01	0.13	0.18**											
4. Agreeableness	4.24	1.05	0.14*	0.28**	0.89**										
5. Conscientiousness	5.25	0.79	− 0.17**	0.01	0.62**	0.56**									
6. Extraversion	5.01	0.84	0.35**	0.07	0.16*	0.10	− 0.15*								
7. Emotional Stability	4.49	0.93	− 0.08	0.04	0.75**	0.77**	0.82**	− 0.05							
8. Openness	5.17	0.76	0.21**	0.12	0.62**	0.54**	0.21**	0.37**	0.25**						
9. Likability Prediction	29.09	30.43	0.20**	0.21**	0.80**	0.75**	0.48**	0.09	0.62**	0.52**					
10. Predicted Agreeableness	4.25	0.83	0.22**	0.33**	0.75**	0.80**	0.43**	0.07	0.60**	0.46**	0.90**				
11. Predicted Conscientiousness	5.25	0.59	− 0.16*	0.03	0.50**	0.44**	0.72**	− 0.21**	0.67**	0.11	0.64**	0.56**			
12. Predicted Extraversion	5.02	0.63	0.44**	0.10	0.10	0.08	− 0.20**	0.70**	− 0.06	0.25**	0.24**	0.16*	− 0.14*		
13. Predicted Emotional Stability	4.49	0.70	− 0.07	0.06	0.65**	0.63**	0.67**	− 0.08	0.77**	0.20**	0.76**	0.76**	0.85**	− 0.05	
14. Predicted Openness	5.17	0.63	0.28**	0.14*	0.52**	0.46**	0.11	0.23**	0.19**	0.79**	0.68**	0.58**	0.19**	0.40**	0.24**

N = 226. *M* and *SD* represent mean and standard deviation, respectively.

*Indicates *p* < 0.05. **Indicates *p* < 0.01.

Such links are not necessarily problematic, as they represent actual phenomena. Studies show that both actual and perceived personality correlate with profession, gender, age, and likability^35,36^. Women, for example, tend to be both: more agreeable than men^37^ and perceived as such^7^. People with desirable personalities are more likable, *and* likable people are perceived to have desirable personalities (i.e., “personality halo effect”)^38^. Yet, such links also imply that it is sufficient to predict demographics and likability to estimate—with some accuracy—perceived personality.

Could models predict perceived personality *beyond* what is explained by likability and demographics? To answer this question, we regress the human perceptions of each of the personality traits against public figures’ likability and demographics. The residuals of these models represent perceived personality traits cleaned of the influence of these variables. Next, we predict these residuals from public figures’ names’ embeddings using Ridge regression.

The results presented in Fig. 1 (red bars) show that GPT-3 can accurately predict perceived personality even when controlling for demographics and likability. The accuracy decreased but remained very high, ranging from r = 0.53 for emotional stability to r = 0.70 for extraversion, which translates into Cohen’s d range of d = 1.25 (very large effect) to d = 1.96 (very large effect). The raw (uncorrected) accuracies range from r = 0.43 to r = 0.61. To put those results in perspective, consider the following well-known diagnostic accuracies: The accuracy of dental X-rays when detecting between-tooth cavities equals r = 0.43; the accuracy of ultrasound results when detecting deep venous thrombosis equals r = 0.60^34^. In other words, even when controlling for demographics and likability, name embeddings allow for diagnosing public figures’ perceived personalities, as widely used medical diagnostic tools allow for diagnosing dental cavities or venous thrombosis. For further comparison, the accuracy of models employing people’s language to predict their own self-reported Big Five scores is about r = 0.40^39^.

The regression models trained here can be further interpreted, as they span GPT-3’s semantic space filled with interpretable words and concepts. For example, the line defined by the regression model predicting perceived extraversion stretches from the edge of semantic space occupied by public figures perceived to be the most introverted to the edge occupied by those perceived to be the most extraverted. To further interpret the models, we map the location of 525 person-descriptive adjectives obtained from^40^ on these regression lines. (Or, in other words, we computed the predicted scores for these adjectives.)

The adjectives maximizing and minimizing the models’ predictions can be found in Table 3 (scores for all 525 adjectives are at https://osf.io/854w2). Those results are highly congruent with the definitions of the Big Five personality traits. For example, adjectives at the bottom of the extraversion scale include “quiet,” “lonely,” “depressed,” “boring,” “lonesome,” “thoughtful,” “withdrawn,” “soft-spoken,” “philosophical,” and “thinking”; while those on top include “entertaining,” “hilarious,” “lively,” “glamorous,” “comical,” “sexy,” “energetic,” “playful,” and “good-humored.” It seems that public figures’ humor, instead of their sociability, is the most salient cue of extraversion to laypeople.

**Table 3:** The 10 person-descriptive adjectives maximizing and minimizing the predictions of a model trained to predict human perceptions.

	Person descriptive adjectives	
	Bottom (ascending)	Top (descending)
Agreeableness	Corrupt Evil Controversial Violent Abusive Jealous Insulting Dishonest Terrible Intimidating	Warm-Hearted Kind-Hearted Compassionate Affectionate Adorable Gentle Good-Natured Cute Lovable Caring
Conscientiousness	Irresponsible Incompetent Alcoholic Messy Disorganized Sloppy Embarrassing Unstable Troubled Disgusting	Sensible Smart Businesslike Intelligent Efficient Punctual Admirable Wise Respectable Athletic
Emotional Stability	Irresponsible Incompetent Alcoholic Messy Disorganized Sloppy Embarrassing Unstable Troubled Disgusting	Gracious Warm-Hearted Straightforward Wise Sensible Thankful Appreciative Admirable Cheerful Gentle
Extraversion	Quiet Lonely Depressed Boring Lonesome Thoughtful Withdrawn Soft-Spoken Philosophical Thinking	Entertaining Hilarious Lively Glamorous Comical Sexy Energetic Playful Good-Humored Cocky
Openness	Conservative Narrow-Minded Closed-Minded Corrupt Terrible Unfair Prejudiced Stupid Incompetent Unsympathetic	Fashionable Creative Artistic Inspirational Entertaining Expressive Imaginative Romantic Adventurous Glamorous
Likability	Evil Corrupt Terrible Disgusting Awful Guilty Violent Hostile Incompetent Bad	Warm-Hearted Kind-Hearted Inspirational Compassionate Gracious Thoughtful Appreciative Respectful Sentimental Grateful

Full lists at https://osf.io/854w2.

Interestingly, those lists correctly captured behavioral correlates of personality. The regression models were trained on human responses to a 10-item personality questionnaire that never mentioned alcohol. Yet, it ranked “alcoholic” as the third (out of 525) adjective most characteristic of low perceived conscientiousness, the third adjective most characteristic of low perceived emotional stability, and the 42nd adjective most characteristic of low agreeableness. This is consistent with past research findings, which linked alcohol addiction with low conscientiousness, low agreeableness, and low emotional stability^41^. Moreover, the results reflect the correlation between the likability of a public figure and the desirability of their perceived personality (i.e., “personality halo effect”). For example, “corrupt” was associated with undesirable (low) levels of agreeableness, emotional stability, and openness.

## Discussion

Our results indicate that public figures’ perceived personality can be accurately predicted from their names’ location in GPT-3’s semantic space. Our models remained accurate even when controlling for public figures’ demographics and overall likability. Moreover, the models showed high face validity as revealed by the examination of public figures predicted to score at the top/bottom of each of the traits, as well as the personality-descriptive adjectives occupying the models’ extremes.

These findings have multiple implications. First, they show that LLMs’ semantic spaces can be used to study and approximate people’s personality perceptions. This could be of interest to researchers and practitioners across disciplines ranging from political psychology to organizational behavior. Second, the research expands our understanding of word embeddings, which bear some similarity to human semantic memory^31^. They are known to encode words’ meanings^42^, including information about group stereotypes^43–45^. Our results show that they also capture individual differences, like individuals’ perceived personality traits. Our studies add to the growing body of social science research utilizing LLMs. For example, recent studies have found that LLMs can predict the directional relationships between ideological attitudes^46^, approximate the voting choices of different social groups^47^, and mirror human behavior in economic games^48^ and reasoning tasks^49^, as well as pass theory of mind tests^50^.

Our studies focused on predicting the perceived personality of public figures with sufficient presence in the sample used to train GPT-3. Yet, a similar approach could be used to measure the perceived personality of people absent from the training data. Given a sample of text describing an individual, one could estimate its location in the model’s semantic space and convert it into perceived personality using regression models trained on public figures. Another limitation of our approach is that it requires collecting human ratings to train regression models. Yet, given the alignment between our models and personality-descriptive adjectives, it is likely that similar results could be achieved without collecting human ratings. Instead, one could predict public figures’ perceived personality by estimating their distance from personality-descriptive adjectives or by asking generative language models to describe a person using person-descriptive adjectives, as we did in our follow-up study^51^. People similar to “outgoing” and dissimilar to “shy,” for example, could be classified as extraverted. Finally, models’ predictions are focused on the period reflected in the training data. For example, if people changed their mind about a given public figure, it would take until the next model training cycle for this change to be reflected in the embeddings.

The feasibility of automated extraction of perceived traits exposes a potential privacy threat^52^. Word embeddings may contain information about traits that the target would prefer to keep private. Even if there are no explicit cues in the training data, the models may still be able to extract intimate information. This mirrors privacy threats pertaining to other data types. For example, people are not very accurate when predicting others’ intimate traits from their facial images or Facebook Likes and thus do not perceive such data as overly sensitive. Yet, computer algorithms achieve high accuracy when extracting personality, political orientation, and even sexual orientation from such data sources^52–54^. The current results show that the impression of intimate traits of public figures can be easily extracted from widely available LLMs. As the collective impression of an individual often correlates with their actual traits, this could amount to a potential threat to privacy^17,55^.

### Supplementary Information

Supplementary Figures.

## Data Availability

The data and code that support the findings of this study are available at: https://osf.io/854w2.
