# Supplementary material for: Large language models know how the personality of public figures is perceived by the general public
Source: Sci Rep. 2024 Mar 20;14:6735. doi: 10.1038/s41598-024-57271-z (PMC10954708; doi:10.1038/s41598-024-57271-z)
Supplement: Supplementary file 1 — Supplementary Figures. [file 41598_2024_57271_MOESM1_ESM.docx]

# Supplementary Materials for

Large Language Models Know How the Personality of Public Figures Is Perceived by the General Public

Xubo Cao (xcao@stanford.edu), Michal Kosinski^1^

**Inter-rater agreement.** The intraclass correlation coefficient (ICC) was computed by taking the first k ratings for each public figure and calculating the shared variance proportion between raters. Targets receiving fewer than k ratings were excluded. The results depicted in Figure S1 illustrate that the ICC increases rapidly with the number of individual ratings and plateaus at approximately 10 ratings. Consequently, public figures with fewer than 10 ratings were omitted from the analysis. The resultant average inter-rater reliability was 0.829. The study’s findings were consistent even when applying lower inclusion thresholds.

**
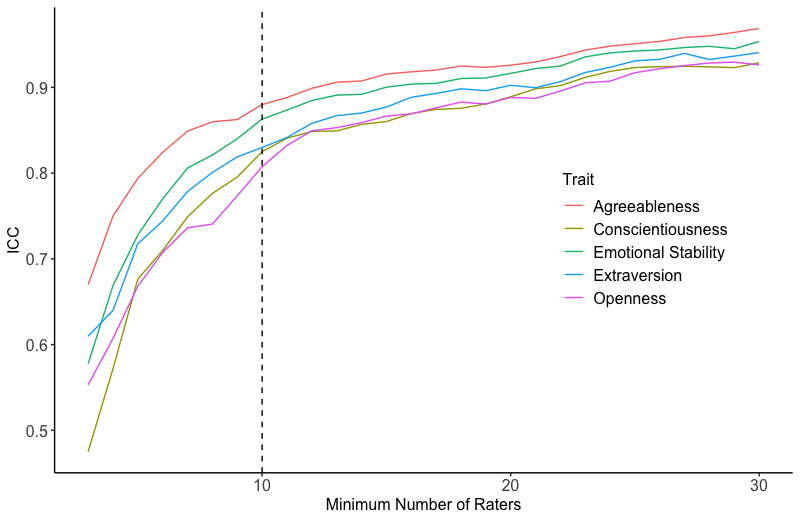
**

**Fig. S1. ICC as a function of the number of raters.**

# Prediction Error as a Function of Wikipedia Pageviews. Our method’s validity hinges on the availability of information about the target individual within the language model’s training corpus. Consequently, we hypothesized that the model’s performance would correlate with the target’s level of fame. To explore this, we conducted a supplementary analysis, plotting the model’s predictive accuracy against the target’s Wikipedia pageviews—a measure of fame. Figure S2 demonstrates the congruence of the model’s predictions with human assessments by depicting profile similarity for each target, quantified as the Pearson correlation coefficient between the model’s predictions and human ratings across the five traits. This correlation is plotted against the log-transformed Wikipedia pageviews.

# The results disclose a positive correlation between the model’s accuracy in predicting profile patterns and the target’s Wikipedia pageviews. These findings suggest that the model is more precise for well-known individuals, which aligns with expectations, considering that such individuals are more frequently mentioned online, thereby featuring more prominently in the model’s training corpus.


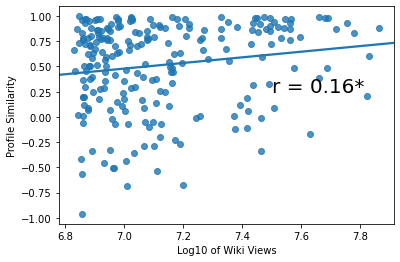


**Fig. S2. Profile similarity between model predictions and human ratings as a function of Wikipedia pageviews.**

**Model Performance Using Ada Engine.** GPT-3 offers various engines tailored to specific user needs. For instance, the DaVinci engine, while more capable, is also slower and costlier compared to the Ada engine. The DaVinci engine produces embeddings with 12,288 dimensions, whereas the Ada engine generates embeddings with 1,024 dimensions. In this analysis, we assess the model’s performance when using Ada and DaVinci embeddings.


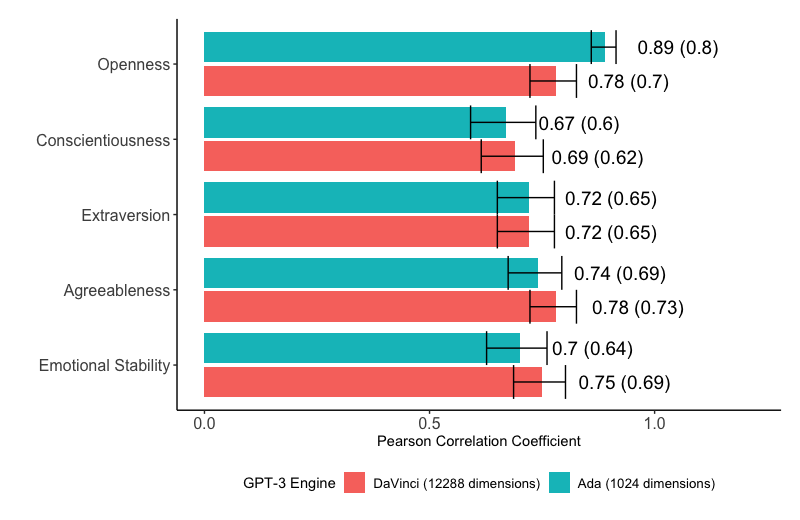


**Fig. S3.** The model’s accuracy at predicting public figures’ perceived personality using different GPT-3 engines. Values in parentheses represent raw accuracy (uncorrected for attenuation). All correlations are significant at the p<.001 level.
